# Establishment of Transient Expression and Hairy Root Induction Systems in *Allium mongolicum*

**DOI:** 10.3390/plants15121799

**Published:** 2026-06-11

**Authors:** Zhenyu Cao, Zhongren Yang

**Affiliations:** College of Horticulture and Plant Protection, Inner Mongolia Agricultural University, Hohhot 010018, China; 15049762270@163.com

**Keywords:** *Allium mongolicum*, hairy root induction, transient expression, vacuum impregnation, subcellular localization

## Abstract

*Allium mongolicum* Regel is a valuable desert plant, yet its functional genomic research is hindered by low genetic transformation efficiency due to monocot regeneration recalcitrance and dense tissue structures. This study established a dual genetic transformation platform for *A. mongolicum*, encompassing in vivo transient expression and stable hairy root induction. By evaluating various infiltration methods, vacuum impregnation was identified as the optimal transient strategy and was successfully applied to localize the candidate proteins AmJAZ2 and AmSWEET14 to the nucleus and plasma membrane, respectively. For stable transformation, shortened stems were utilized as the optimal target explants. An orthogonal experimental design was employed to optimize key parameters, including *Agrobacterium* density, acetosyringone concentration, vacuum parameters, and co-cultivation duration. GFP fluorescence and PCR analysis confirmed the stable integration and expression of the transgene in the induced hairy roots. In conclusion, this study establishes preliminary, species-specific genetic transformation protocols for *A. mongolicum*, providing a baseline technical reference that may support subsequent exploratory research on the molecular biology and secondary metabolism of this species.

## 1. Introduction

*Allium mongolicum* Regel is an important wild *Allium* species widely distributed in arid and semi-arid regions [1]. As a typical xerophyte, it exhibits extraordinary environmental adaptability to survive in harsh sandy habitats, playing a critical role in ecological restoration and desertification control [2]. Furthermore, *A. mongolicum* is rich in characteristic secondary metabolites, such as sulfur-containing volatiles, flavonoids, and polysaccharides [3]. These compounds not only confer significant broad-spectrum biological activities, including antioxidant properties, but also demonstrate immense application potential in the livestock and food industries (e.g., serving as feed additives to improve meat flavor) [4].

Recently, research on *A. mongolicum* has progressively advanced from fundamental resource surveys and tissue culture to molecular biology and metabolomics. With the rapid advancement of high-throughput sequencing technologies, transcriptomic and genomic data of *A. mongolicum* have been continuously deciphered, leading to the identification of numerous candidate functional genes related to stress tolerance and secondary metabolism [5]. However, despite its immense agronomic and scientific potential, systematically elucidating the genetic basis of these traits remains a core challenge. Specifically, the lack of an efficient and stable gene function verification system confines current exploration largely to preliminary bioinformatic predictions. This limitation has constrained the further development of functional genomics in *A. mongolicum.*

In the field of plant genetic transformation, monocotyledonous plants (especially those in the Allium genus) have long been recognized as highly recalcitrant to *Agrobacterium* infection [6]. This recalcitrance stems primarily from two factors. First, monocots lack a typical vascular cambium with high meristematic potential, and their cell walls tend to undergo early lignification and silicification during development. This results in an extremely low tissue regeneration capacity, making it difficult to form competent cells receptive to T-DNA integration [7]. Second, long-term adaptation to extreme desert habitats has driven *A. mongolicum* to evolve highly dense anatomical structures, as well as a well-developed surface cuticle and a thick wax layer [8]. While these specialized physiological traits confer robust environmental adaptability, they also act as formidable natural physical and biochemical barriers that impede *Agrobacterium* attachment, tissue penetration, and T-DNA delivery. Although recent progress has been made in *Agrobacterium*-mediated genetic transformation, transient expression, and hairy root induction in related economically important Allium crops, such as onion, Welsh onion, and garlic [9,10], transformation studies targeting the wild desert plant *A. mongolicum* have not yet been reported. The absence of standardized transient expression and stable transformation protocols has limited molecular biology and metabolic research in this species.

To support functional genomics research in *A. mongolicum*, the development of adapted protocols for both transient expression and stable transformation is highly desirable. At the transient expression level, determining the subcellular localization of target proteins is a fundamental prerequisite for elucidating their biological functions, molecular interaction networks, and regulatory pathways [11]. *Agrobacterium*-mediated transient transformation has become a routine technique in modern plant biology due to its simplicity and rapid expression. However, confronting the dense leaf structure of *A. mongolicum*, traditional syringe infiltration often proves ineffective. Therefore, there is a pressing need to introduce and optimize physically assisted strategies, such as vacuum impregnation, to effectively overcome tissue and physiological barriers, thereby achieving in vivo protein localization imaging with a high signal-to-noise ratio and minimal tissue damage [12,13].

At the stable transformation level, considering the objective reality of the inherent recalcitrance of whole-plant regeneration among Liliaceae species, the *Agrobacterium* rhizogenes-mediated hairy root induction system offers an ideal breakthrough solution. Hairy roots possess excellent characteristics, including rapid and highly branched growth, and the ability to proliferate indefinitely in vitro without the need for exogenous plant hormones. More importantly, they enable the stable integration and continuous expression of exogenous genes within the host genome [14,15]. For *A. mongolicum*, the development of a reproducible hairy root transformation protocol offers several potential advantages. On the one hand, it bypasses the hurdles of whole-plant tissue regeneration, providing a culture model that could theoretically be adapted in the future for gene editing configurations (such as CRISPR/Cas9) or the long-term functional validation of stress-related genes [16,17]. On the other hand, hairy roots represent a potential culture system that may facilitate subsequent investigations into the biosynthetic pathways and regulatory mechanisms of high-value secondary metabolites unique to *A. mongolicum*, such as flavonoids, polyphenols, and characteristic organosulfur flavor compounds [18,19].

To address the aforementioned research gaps, this study established and optimized two genetic transformation systems for *A. mongolicum*: in vivo transient expression and stable hairy root induction. First, by evaluating various physical infiltration methods and receptor organs, a vacuum-assisted transient transformation system was developed for leaf tissues and successfully applied to characterize the subcellular localization of the candidate genes *AmJAZ2* and *AmSWEET14*. Second, an *Agrobacterium* rhizogenes-mediated hairy root induction system was established by targeting the shortened stem region. Key parameters, including bacterial density (OD_600_), acetosyringone (AS) concentration, vacuum treatment, and co-cultivation duration, were optimized using an orthogonal experimental design. The stable integration and expression of the exogenous gene in the hairy roots were verified via GFP fluorescence observation and PCR analysis. This study achieves a successful technical adaptation of genetic transformation in *A. mongolicum* and provides an optimized, standardized protocol, providing a primary technical reference for future academic exploration into the functional genomics and secondary metabolism of this species.

## 2. Results

### 2.1. Optimization of Agrobacterium-Mediated Transformation for Subcellular Localization in A. mongolicum

**Figure 1:**
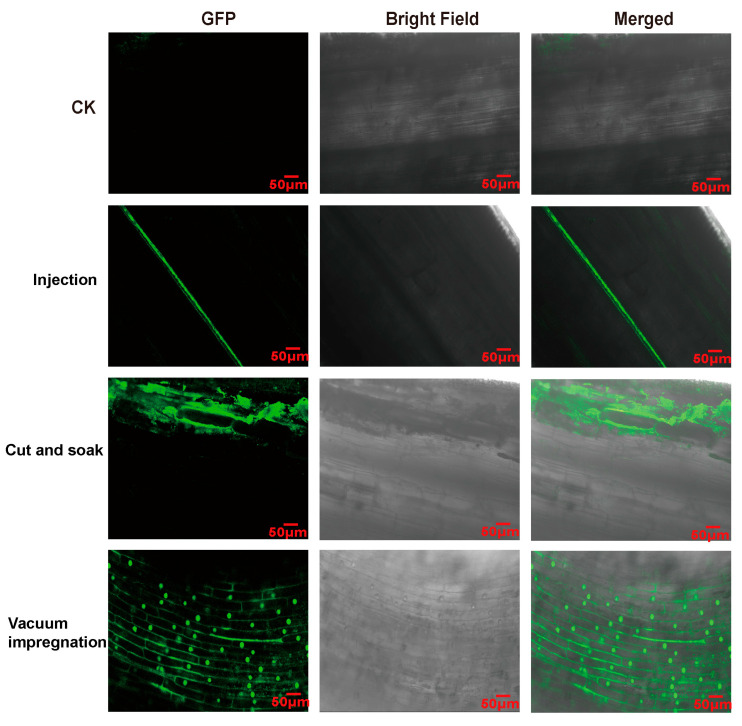
Comparison of different *Agrobacterium*-mediated infiltration methods for transient expression in *A. mongolicum.* Transient expression of the 35S:GFP construct was evaluated using three infiltration techniques. CK: Negative control without Agrobacterium infection; Injection: Direct syringe infiltration into the leaf tissue; Cut and soak: Tissues were wounded and immersed in *Agrobacterium* suspension; Vacuum impregnation: Tissues were subjected to vacuum pressure in *Agrobacterium* suspension. From left to right: GFP fluorescence (GFP), Bright field, and Merged images. Scale bars = 50 μm.

To establish a robust subcellular localization system for *A. mongolicum*, three different *Agrobacterium*-mediated infiltration methods—injection, cut and soak, and vacuum impregnation—were evaluated using a GFP reporter gene. As shown in Figure 1, no fluorescence was detected in the negative control (CK). The injection method resulted in highly localized signals restricted to the injection track, likely due to the structural density of *A. mongolicum* tissues preventing the efficient spread of the *Agrobacterium* suspension. Similarly, the cut and soak method yielded uneven fluorescence primarily restricted to the wound sites, with overall low transformation efficiency.

In contrast, vacuum impregnation significantly enhanced the transformation efficiency, as evidenced by the most intense and uniform green fluorescence across a large number of cells. Under confocal microscopy, the GFP signal was clearly visible in both the cytoplasm and the nucleus (presenting as a distinct, uniform nuclear signal), demonstrating high-level transient expression of the exogenous protein. These findings indicate that vacuum impregnation is the optimal approach for building a high-efficiency subcellular localization and transient expression platform in *A. mongolicum*.

### 2.2. Evaluation of Different A. mongolicum Tissues for Transient Expression

**Figure 2:**
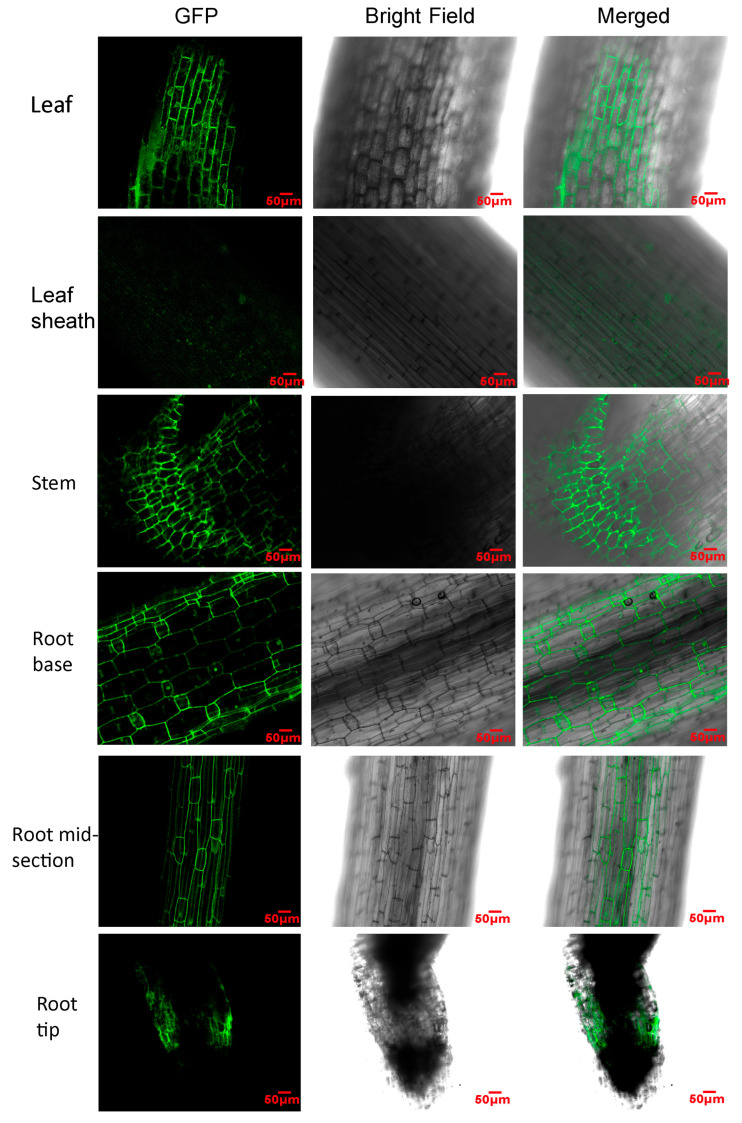
Transient expression efficiency of GFP in different tissues of *A. mongolicum*. Various tissues, including the leaf, leaf sheath, stem, base of the root, middle of the root, and root tip, were infected with *Agrobacterium* harboring the 35S:GFP construct via vacuum impregnation. The images demonstrate the varied suitability of different plant parts for subcellular imaging. From left to right: GFP fluorescence (GFP), bright-field images (Bright Field), and merged images (Merged). Scale bars = 50 μm.

To identify the most suitable tissue for subcellular localization imaging, various developmental parts of *A. mongolicum*—including the leaf, leaf sheath, stem, root base, root mid-section, and root tip—were subjected to *Agrobacterium*-mediated transient transformation using the optimized vacuum impregnation method. As illustrated in Figure 2, significant variations in transformation efficiency and fluorescence intensity were observed among the different tissues. Robust and uniform GFP signals were observed across various tissues, including leaves, shortened stems, and root bases. Notably, leaf tissues were prioritized for subsequent subcellular localization assays due to their highly regular cellular arrangement and clear cell differentiation, which facilitated a more precise interpretation of subcellular signal distribution compared to the densely packed cells in stem tissues. In particular, the leaf tissue proved to be the most ideal material; its regular cellular arrangement and excellent light transmittance resulted in a high signal-to-noise ratio, allowing for clear visualization of the cell outlines. Distinct fluorescence was also observed in the stem and the middle of the root, though slightly less uniform than in the leaves. Conversely, minimal to no GFP signal was detected in the leaf sheath. While the root tip exhibited some localized fluorescence, its dense tissue structure severely reduced light penetration in the bright field, making it less favorable for high-resolution imaging. Collectively, these results demonstrate that the leaf of *A. mongolicum* serves as the optimal explant for the subcellular localization system.

### 2.3. Optimization of Parameters for Transient Transformation in A. mongolicum

Having identified vacuum impregnation and leaf tissue as the optimal method and explant, we further optimized two critical parameters influencing *Agrobacterium* infection—bacterial suspension concentration (OD_600_) and acetosyringone (AS) concentration—using an orthogonal experimental design. As detailed in Table 1, the transformation efficiencies varied notably among the nine treatments, ranging from 34.44% to 75.56%.

Range analysis (R) was performed to evaluate the magnitude of each factor’s effect on transformation efficiency. The R-value for OD_600_ (28.15%) was substantially higher than that for AS concentration (2.60%), indicating that the bacterial concentration is the primary determinant of transient transformation efficiency in *A. mongolicum*. Notably, as the OD_600_ increased from 0.4 to 0.8, the average transformation efficiency (k-value) sharply decreased from 65.18% to 37.04%. This decline suggests that an excessively high concentration of *Agrobacterium* may trigger a hypersensitive response leading to tissue necrosis, or exhibit toxicity to the plant tissues. Conversely, variations in AS concentration within the tested range exerted a marginal effect on efficiency. Based on the k-values and direct experimental observations, Treatment 3 yielded the highest efficiency. Specifically, the combination of an OD_600_ of 0.4 and an AS concentration of 200 μM resulted in a peak transformation efficiency of 75.56%. Consequently, this combination was established as the standard protocol for subsequent subcellular localization analyses in *A. mongolicum*.

Furthermore, the multi-factor ANOVA via the GLM framework revealed a highly significant interaction between OD_600_ and AS concentration (*p* < 0.001). This significant interaction demonstrates that the efficacy of AS in facilitating T-DNA transfer is highly dependent on the initial bacterial titer. Specifically, a higher AS concentration (200 μM) dramatically boosted transformation efficiency only when paired with a lower bacterial density (OD_600_ = 0.4), whereas at higher bacterial densities (OD_600_ = 0.6 or 0.8), increasing AS failed to confer a similar advantage, likely due to premature bacterial overgrowth or hypersensitive tissue necrosis.

### 2.4. Co-Localization of AmJAZ2 and AmSWEET14

To test the biological applicability of the optimized transient expression system, two genes associated with stress-responsive and metabolic pathways—*AmJAZ2* and *AmSWEET14*—were selected as preliminary candidate examples. *AmJAZ2* is linked to the jasmonate signaling network that frequently participates in environmental adaptation, while *AmSWEET14* belongs to a sugar transporter family often involved in carbohydrate partitioning.

Before experimental validation, the structural architectures and theoretical subcellular localizations of both candidate proteins were comprehensively analyzed via in silico bioinformatics profiling (Supplementary Figure S1). Conserved domain analysis using the NCBI Conserved Domain Database (CDD) revealed that AmSWEET14 (271 aa) possesses two classic duplicated MtN3_slv domains, which structurally align with the MtN3/saliva sugar transporter superfamily (Figure S1A). Concurrently, the computational algorithm Plant-mPLoc strongly predicted a plasma membrane destination for AmSWEET14 (Figure S1C). Regarding AmJAZ2 (222 aa), domain parsing identified a standard TIFY superfamily domain (residues 101–142) alongside a highly conserved C-terminal Jas_motif (residues 177–203), representing the functional evolutionary fingerprints of nuclear-localized jasmonate-ZIM domain repressors (Figure S1B). This structural classification was further supported by Plant-mPLoc modeling, which uniquely assigned AmJAZ2 to the nucleus (Figure S1D).

**Figure 3:**
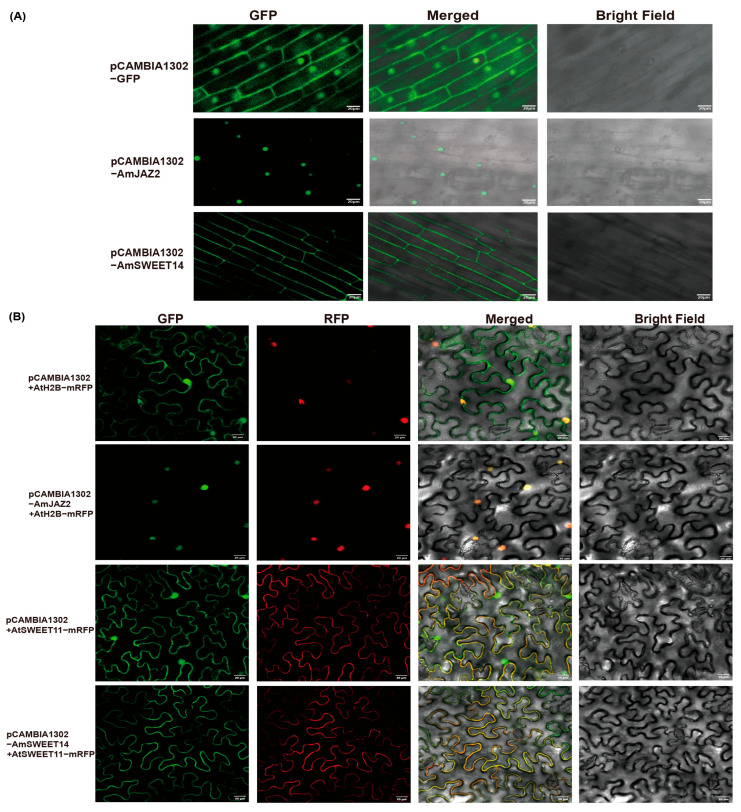
Subcellular localization validation of AmJAZ2 and AmSWEET14. (**A**) Transient expression in *A. mongolicum* leaves via the optimized vacuum impregnation method. pCAMBIA1302-GFP served as the control. (**B**) Co-localization analysis in *N. benthamiana* leaves. AmJAZ2-GFP was co-expressed with the nuclear marker AtH2B-mRFP, and AmSWEET14-GFP was co-expressed with the plasma membrane marker AtSWEET11-mRFP. Scale bars = 20 μm. From left to right: GFP channel (green), RFP channel (red, only for (**B)**), Merged images, and Bright Field images.

Supported by these robust theoretical baselines, the encoded proteins were transiently expressed in both *A. mongolicum* and *N*. *benthamiana* leaves to determine their spatial distribution. In the *A. mongolicum* transient expression system (Figure 3A), the signals from the empty vector (pCAMBIA1302-GFP) were distributed throughout the nucleus and cytoplasm. In contrast, the AmJAZ2-GFP fusion protein exhibited exclusive fluorescence within the nucleus, appearing as distinct punctate signals. Meanwhile, the AmSWEET14-GFP protein was primarily localized to the cell periphery, suggesting a plasma membrane distribution. Given the current technical constraints of achieving high-efficiency dual-plasmid co-transformation directly in this non-model species, the specific subcellular localizations in *A. mongolicum* were initially inferred from these distinct GFP spatial patterns. To structurally support and validate these inferences, formal co-localization assays were subsequently performed in the well-established *N. benthamiana* epidermal cell system (Figure 3B). The green fluorescence of AmJAZ2-GFP perfectly overlapped with the red fluorescence of the nuclear marker AtH2B-mRFP, confirming its nuclear localization. Similarly, the green fluorescence of AmSWEET14-GFP was precisely co-localized with the plasma membrane marker AtSWEET11-mRFP. These findings not only clarify the subcellular sites of AmJAZ2 and AmSWEET14 but also demonstrate that the established *A. mongolicum* system serves as a workable and biologically relevant platform for inferring the subcellular distribution of diverse candidate proteins.

### 2.5. Preliminary Exploration and Optimization of the Hairy Root Induction System

To establish a robust hairy root induction platform for *A. mongolicum*, the effects of different *Agrobacterium rhizogenes* strains and explant types on transformation efficiency were systematically evaluated.

**Figure 4:**
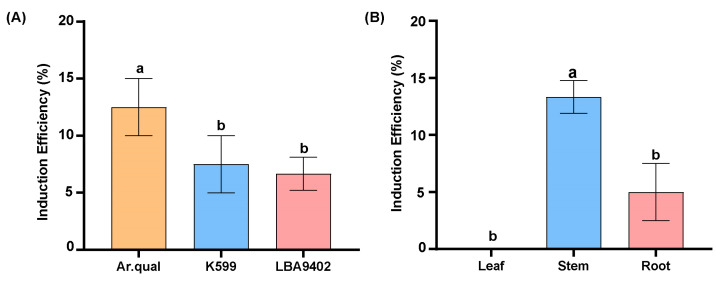
Effects of bacterial strains and explant types on hairy root induction in *A. mongolicum.* (**A**) Evaluation of hairy root induction efficiency using three *A. rhizogenes* strains (Ar.qual, K599, and LBA9402). (**B**) Comparison of induction efficiency among different explant types (leaf, stem, and root). Leaf explants (0% efficiency) were included in the statistical analysis. Data are presented as mean ± SD of three biological replicates. Different lowercase letters above the bars indicate significant differences according to one-way ANOVA followed by Tukey’s HSD test (*p* < 0.05).

Regarding the choice of bacterial strains (Figure 4A), all three tested *A. rhizogenes* strains (Ar.qual, K599, and LBA9402) were capable of inducing hairy roots, albeit with varying efficiencies. Ar.qual exhibited the highest infectivity, yielding a peak induction rate of 12.50%, followed by K599 (7.50%). LBA9402 showed the lowest induction capacity (6.67%). These results suggest that Ar.qual possesses superior compatibility with *A. mongolicum* tissues.

The type of explant also significantly influenced the induction process (Figure 4B). Among the tested tissues, the shortened stem proved to be the most responsive explant, achieving the highest transformation efficiency of 13.33%, which was statistically significantly higher than that of both root and leaf tissues (*p* = 0.0012). In contrast, root explants showed a substantially lower induction rate (4.17%). Notably, no hairy root formation was observed in leaf explants (0.00%) across multiple trials, indicating high resistance to *A. rhizogenes* infection in this tissue. Collectively, the combination of the Ar.qual strain and shortened stem explants was identified as the optimal strategy for hairy root induction in *A. mongolicum.*

### 2.6. Fine-Tuning of Parameters for A. rhizogenes-Mediated Hairy Root Induction

To further maximize the efficiency of the hairy root induction system, an orthogonal experimental design was implemented to optimize the bacterial concentration (OD_600_) and AS concentration. As shown in Table 2, the induction efficiencies varied from 5.00% to 18.33% across the nine treatment combinations.

Range analysis (R) indicated that OD_600_ (R = 8.61%) exerted a more substantial influence on hairy root induction compared to AS concentration (R = 5.00%). This identifies bacterial density as the primary factor determining the success of *A. rhizogenes* infection in *A. mongolicum*. A clear downward trend in induction efficiency was observed with increasing OD_600_ (k-values decreased from 14.72% to 6.11%), suggesting that a lower bacterial density (0.3) helps mitigate excessive tissue damage and promotes stable transformation. Furthermore, an AS concentration of 100 µM yielded the highest average efficiency among the tested levels.

The multi-factor ANOVA for the hairy root induction system (Table 2) demonstrated a highly robust and distinct statistical behavior compared to the transient platform. Both OD_600_ and AS concentration exerted highly significant individual main effects on the induction rate (*p* < 0.001), with a clear peak efficiency of 18.33 ± 1.44% locked at OD_600_ = 0.3 paired with 100 μM AS. Interestingly, the interaction term between bacterial density and AS concentration was statistically non-significant (*p* > 0.05). This lack of interactive synergy indicates that the two factors influence root organogenesis via independent physiological vectors, where increasing the AS dosage beyond 100 μM triggers a generalized, parallel suppression of induction efficiency across all tested bacterial concentrations.

In summary, the combination in Treatment 1 (OD_600_ = 0.3, AS = 100 µM) was identified as the optimal protocol, achieving a peak induction efficiency of 18.33%. These optimized parameters provide a reliable foundation for the stable and reproducible generation of transgenic hairy roots and subsequent functional genomic studies in *A. mongolicum*.

### 2.7. Optimization of Physical Assistance and Co-Cultivation Duration for Hairy Root Induction

Building upon the optimized bacterial concentration and chemical inducers (i.e., AS), the influence of physical assistance (vacuum intensity) and environmental parameters (co-cultivation time) on *A. mongolicum* hairy root transformation was further investigated. As detailed in Table 3, the induction efficiencies varied significantly among the treatments, reaching a maximum of 20.83%.

Range analysis (R) indicated that co-cultivation time (R = 6.94%) had a slightly greater impact on induction efficiency than vacuum intensity (R = 5.55%). An analysis of the k-values revealed that the induction efficiency followed a bell-shaped curve relative to the duration of co-cultivation, peaking at 72 h (k2 = 15.55%). This suggests that while sufficient co-cultivation facilitates T-DNA integration, excessive exposure (up to 96 h) may lead to tissue browning and necrosis due to *Agrobacterium* overgrowth.

Regarding vacuum intensity, −0.06 MPa was identified as the optimal pressure level (k2 = 15.28%). Appropriate negative pressure likely enhances the penetration of *Agrobacterium* into the deeper tissues of *A. mongolicum*, whereas excessive vacuum (e.g., −0.08 MPa) significantly impaired the induction capacity, possibly due to mechanical damage to the explant cells. Overall, Treatment 5, combining 72 h of co-cultivation with a vacuum intensity of −0.06 MPa, yielded the highest induction efficiency of 20.83%.

The optimization of physical parameters (Table 3) was statistically evaluated via a multi-factor ANOVA, which revealed a highly significant interactive effect (*p* < 0.001) between the co-cultivation duration and vacuum pressure. This pronounced interaction indicates that successful T-DNA delivery relies on a delicate physiological equilibrium between bacterial penetration and host tissue viability. The data demonstrated that a moderate vacuum of −0.06 MPa coupled with a 72-h co-cultivation yielded the absolute peak efficiency (20.83 ± 1.44%). Notably, exposing the tissues to a harsher vacuum (−0.08 MPa) resulted in a progressive collapse of induction efficiency as the co-cultivation time extended, highlighting severe mechanical and localized hypersensitive tissue necrosis. Conversely, while −0.06 MPa facilitated optimal bacterial entry, extending the co-cultivation to 96 h triggered a steep decline in efficiency (8.33%), likely driven by bacterial overgrowth that suffocated the vulnerable stem explants.

Consequently, a feasible and optimized protocol for *A. mongolicum* hairy root induction was successfully established.

### 2.8. Validation and Molecular Verification of Transgenic Hairy Roots

**Figure 5:**
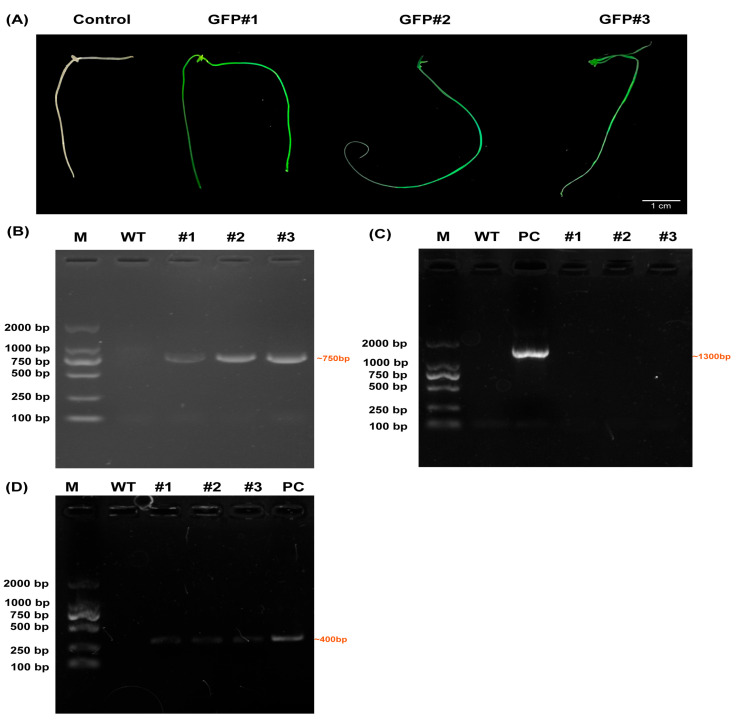
Identification and verification of transgenic hairy roots in *A. mongolicum*. (**A**) Green fluorescence observation of non-transformed control roots and three independent transgenic hairy root lines (GFP#1, GFP#2, GFP#3). Scale bar = 1 cm. (**B**–**D**) PCR verification of genomic DNA extracted from the respective root lines. (**B**) Amplification of the binary vector GFP gene (expected size ~750 bp). (**C**) Counter-screening amplification of the bacterial backbone virD2 gene (expected size ~1.3 kb) to confirm the elimination of residual *A. rhizogenes*. (**D**) Amplification of the Ri-plasmid T-DNA *rolB* gene (expected size ~400 bp) to confirm co-transformation. Lane M: DNA marker; Lane WT: Non-transformed wild-type root (negative control); Lane PC: Pure *A. rhizogenes* culture (positive control); Lanes #1–#3: Three independent GFP-positive hairy root lines.

To confirm the transgenic nature of the hairy roots induced via the optimized protocol, the generated root lines were subjected to fluorescence observation and molecular analysis. As shown in Figure 5A, under a GFP excitation light, the control root (non-transformed) exhibited no background fluorescence. In contrast, three randomly selected independent hairy root lines (GFP#1, GFP#2, and GFP#3) displayed intense and uniform green fluorescence along their entire length, indicating the successful expression of the exogenous GFP reporter gene.

Agarose gel electrophoresis (Figure 5B) revealed that all three GFP-positive hairy root lines (lanes 1–3) yielded specific amplified bands corresponding to the binary vector *GFP* gene. Furthermore, successful co-transformation was confirmed by the positive amplification of the *rolB* gene in these transgenic lines, indicating the expected integration of the Ri-plasmid T-DNA. In contrast, neither *GFP* nor *rolB* bands were detected in the non-transformed control root. Crucially, the counter-screening PCR targeting the bacterial backbone gene *virD2* yielded a distinct band only in the pure *A. rhizogenes* positive control, remaining completely undetectable in the non-transformed control and all three transgenic hairy root lineages. The correlation among visible GFP fluorescence, the presence of both *GFP* and *rolB* transgenes, and the complete absence of the *virD2* bacterial marker provides definitive, unambiguous evidence that both the binary and Ri T-DNAs were successfully and stably co-integrated into the *A. mongolicum* genome, free of bacterial contamination. Collectively, these findings successfully validate the establishment of a reproducible and reliable hairy root induction system for *A. mongolicum.*

### 2.9. Standardized and Workable Protocol for Hairy Root Induction in A. mongolicum

**Figure 6:**
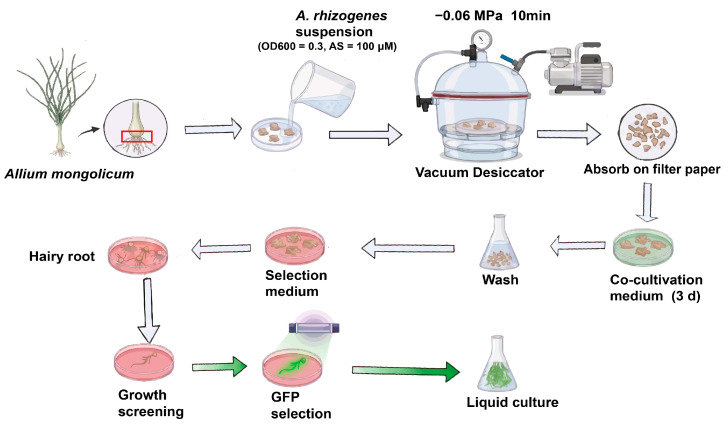
Schematic representation of the optimized *A. rhizogenes*-mediated hairy root induction system for *A. mongolicum.*

Based on the systematic optimization and validation of bacterial strains, explant types, and various physicochemical parameters described above, we finally established a standardized and reproducible protocol for the induction of transgenic hairy roots in *A. mongolicum.* The sequential steps of this optimized workflow are schematically summarized in Figure 6.

Briefly, the process begins with the preparation of shortened stems from healthy *A. mongolicum* plants, which serve as the optimal explants. These explants are immersed in an *A. rhizogenes* suspension adjusted to an optimal density (OD_600_ = 0.3) and supplemented with 100 µM AS. To facilitate the deep penetration of the bacteria into the plant tissues, the mixture is subjected to vacuum infiltration in a desiccator at −0.06 MPa for 10 min. Following infiltration, the explants are blotted dry on sterile filter paper to remove excess bacterial suspension and then transferred to a co-cultivation medium in the dark for 3 days (72 h).

After co-cultivation, the explants are thoroughly washed to eliminate the remaining *Agrobacterium* and placed on a selection medium to induce hairy root emergence. Once the hairy roots develop, robust growth screening and GFP fluorescence visualization are performed to identify true-positive transformants. Finally, the confirmed transgenic hairy roots are excised and transferred into a liquid culture system for rapid proliferation, providing abundant materials for subsequent functional genomic analyses or secondary metabolite investigations.

## 3. Discussion

*Allium mongolicum*, an essential desert plant of the *Allium* genus (*Liliaceae*), possesses significant ecological value for windbreak and sand fixation, as well as considerable economic and medicinal potential due to its unique secondary metabolites. However, long-term adaptation to extreme habitats—characterized by drought and intense ultraviolet radiation—has led to the evolution of dense tissue structures, developed cuticles, and highly lignified cell walls. These physiological barriers, combined with the recalcitrance to regeneration common in Liliaceae species, have historically resulted in extremely low genetic transformation efficiencies, severely hindering functional genomic research at the molecular level. To establish a tailored genetic toolset for this species, this study systematically addressed the challenges of *Agrobacterium* infection in *A. mongolicum*. We have not only established and optimized a high-efficiency transient transformation and subcellular localization system but also successfully constructed a stable and reproducible transgenic hairy root induction platform. The optimization of these protocols provides a primary technical reference that may assist future efforts to explore stress-resistance gene functions, analyze secondary metabolic pathways, or support subsequent molecular breeding research in this species.

In plant transient transformation systems, the choice of physical infiltration method and receptor tissue directly dictates transformation efficiency. This study found that vacuum impregnation significantly outperformed syringe infiltration in *A. mongolicum* tissues. Mechanistically, vacuum impregnation utilizes pressure differentials to evacuate air from tissue interstices, followed by the rapid infusion of the *Agrobacterium* suspension into the intercellular and apoplastic spaces upon the restoration of atmospheric pressure [20]. This non-invasive approach effectively overcomes the limitations of syringe infiltration, such as uneven local diffusion and restricted penetration depth caused by manual pressure. Furthermore, vacuum impregnation significantly minimizes mechanical damage, thereby avoiding oxidative bursts, callose deposition, and programmed cell death typically triggered by needle punctures [21,22]. Research has also indicated that vacuum treatment enhances the colonization density of *Agrobacterium* within the mesophyll intercellular spaces, which in turn improves *Vir* gene activation efficiency and T-DNA delivery rates [23,24]. High-efficiency expression via vacuum impregnation has been reported in the young tissues of *Arabidopsis*, whereas syringe infiltration proved difficult to apply due to tissue fragility [22]. Similarly, vacuum impregnation has demonstrated excellent reproducibility and scalability in species that are traditionally difficult to transform, such as tomato and avocado [25,26].

Regarding receptor tissues, this study identified *A. mongolicum* leaves as the optimal choice for transient transformation, while leaf sheaths and root tips exhibited poor performance. While the dense epidermis acts as a barrier, the underlying spongy mesophyll consists of parenchyma cells with moderate mitotic activity and intercellular spaces, which, once accessed via vacuum impregnation, providing natural entry points for *Agrobacterium*. Moreover, the spongy mesophyll consists of parenchyma cells with moderate mitotic activity, abundant organelles (e.g., endoplasmic reticulum and Golgi apparatus), and high metabolic energy, providing a metabolic context that supported the expression and visualization of exogenous GFP proteins in this study [27]. High-efficiency transient expression in leaves has been similarly achieved in species such as spinach and peony [28,29]. In contrast, leaf sheath cells are highly lignified and silicified, with dense cell walls and sparse plasmodesmata that impede *Agrobacterium* colonization and the movement of expression products [30]. Furthermore, although specialized root-targeting transformation systems have been successfully established in some woody or herbaceous species [31], the root tips of *A. mongolicum* proved less suitable in this study. Their dense vascular structures may impede *Agrobacterium* penetration, or potentially trigger plant defense responses, such as callose deposition or programmed cell death, which hindered efficient transformation in these tissues.

Additionally, precise control of the bacterial suspension density (OD_600_) is critical for balancing T-DNA delivery and host defense responses. The OD_600_ value directly influences the physiological state of *Agrobacterium* and the activation levels of Vir genes. Excessively high OD_600_ values can trigger robust plant immune responses, such as reactive oxygen species bursts, which hinder T-DNA integration [32,33]. Studies in various species, such as industrial hemp and camphor, have consistently identified a specific optimal OD_600_ window [34,35]. Maintaining a consistent bacterial inoculation density is essential to ensure that the expression effects of candidate genes are scientifically comparable.

Beyond technical baseline optimization, the precise subcellular localization of AmJAZ2 and AmSWEET14 provides preliminary spatial insights into their potential physiological roles in *A. mongolicum*. In our transient assays, the empty vector control displayed a generalized distribution throughout the nucleus and cytoplasm, whereas the AmJAZ2-GFP fusion protein exhibited exclusive, punctate signals within the nucleus. This nuclear localization is consistent with its predicted function as a transcriptional repressor in the jasmonate pathway, where it may interact with nuclear-localized transcription factors to modulate stress-responsive or secondary metabolic downstream target genes. Similarly, the distinct distribution of AmSWEET14-GFP at the cell periphery indicates a plasma membrane localization. As a typical desert xerophyte distributed in arid and semi-arid regions, *A. mongolicum* has evolved to survive in harsh sandy habitats. The plasma membrane localization of AmSWEET14 suggests its potential engagement in exporting intracellular sugars to the apoplast, which may facilitate systemic energy redistribution or osmotic adjustment under environmental stress conditions. Taken together, these successful applications suggest that the established transient platform is a biologically relevant tool capable of providing primary structural and spatial clues for functional genomics in this desert species.

However, it must be emphasized that the methodological validation in this current study relies primarily on GFP reporter expression and genomic PCR verification. While these lines of evidence demonstrate successful T-DNA delivery and stable integration, the actual physiological functionality of the system—including its direct impacts on the expression analysis of stress-related endogenous genes or quantitative metabolite profiling in transformed tissues—remains uncharacterized at this stage and should not be overstated. Characterizing these downstream biochemical phenotypes requires further empirical investigation, such as executing quantitative real-time PCR to track transcription networks or conducting mass spectrometry (LC-MS) to resolve metabolic flux alterations in specific overexpressed or silenced lineages. In future investigations, incorporating these complementary molecular and chemical assays across a broader repertoire of target genes will be pursued to comprehensively verify the functional utility and depth of this genetic transformation toolset.

Unlike transient expression, the core of hairy root induction lies in triggering explant cell dedifferentiation and initiating root organogenesis. Regarding the selection of receptor tissues, this study identified the shortened stem at the junction of the bulb and the root system as the optimal target explant for inducing hairy roots in *A. mongolicum*. Anatomically, monocotyledonous Liliaceae plants, as exemplified by members of the *Allium* genus like garlic, typically lack a defined vascular cambium and undergo early cell wall lignification, which collectively confer strong recalcitrance to Agrobacterium infection [36,37]. However, as a member of the *Allium* genus, *A. mongolicum* shares a close phylogenetic relationship and high structural homology with garlic (*A. sativum*). Recent histological studies have definitively shown that the stem disc region of garlic is enriched with soft parenchyma tissue, active meristematic cells, and densely packed vascular strands [10,38]. Consequently, the shortened stem of *A. mongolicum*, which shares anatomical similarities with the stem disc of garlic, was selected as an explant. The observed induction efficiency of 13.33% in this region supports its suitability as a target site for T-DNA integration. In the present study, the strategy of pre-excising the existing roots and leaves to precisely isolate the shortened stem as the explant proved to be highly effective. This mechanical excision not only directly exposed the deep parenchymal target cells to the *Agrobacterium* suspension, eliminating physical barriers and nutrient competition from mature tissues, but also acted as a classic mechanical wounding signal. The excision induced the plant tissue to release large amounts of endogenous phenolic compounds, which acted synergistically with the exogenously applied AS to hyper-activate the *Agrobacterium vir* genes.

Furthermore, at the endogenous hormone level, the IAA/CTK (auxin/cytokinin) ratio in stem segments is more conducive to the initiation of root primordia. The expression of the *Agrobacterium rolB* gene in this context further amplifies the high-auxin effect. This is consistent with empirical studies in Astragalus, garlic, and Verbascum, where stem or stem disc regions were identified as pivotal sites for *Agrobacterium* infection and morphogenesis [39,40].

In terms of co-cultivation duration, this experiment confirmed the existence of an “optimal window.” The primary objective of co-cultivation is to achieve a balance between ensuring effective T-DNA transfer and maintaining plant cell viability. An insufficient duration results in incomplete T-DNA integration, while excessive duration leads to severe tissue browning and cell death. Although the optimal duration varies among species—such as 48 h for chicory *(Cichorium intybus*) [41] and peanut(*Arachis hypogaea*) [42]—a window of 48–72 h remains the gold standard for ensuring high survival and induction rates.

A comparison between the two established systems reveals significant differences in the required bacterial concentrations. Subcellular localization requires a higher OD_600_ (0.4 in this study), whereas the optimal concentration for hairy root induction is considerably lower (0.3 or below). This divergence in parameter selection reflects the fundamental differences in the biological objectives of the two experiments. Subcellular localization aims for high-abundance protein expression within a narrow time window (24–72 h); thus, a higher concentration of *Agrobacterium* is necessary to maximize T-DNA delivery and ensure a high signal-to-noise ratio for microscopic detection (as seen in studies of the BP8-2 protein) [43]. Conversely, hairy root induction seeks stable, long-term genetic transformation. Once even a small number of *Agrobacterium* cells successfully integrate the T-DNA, the cell fate is irreversibly altered. Studies in *Citrullus* and *Calotropis* have confirmed that excessive *Agrobacterium* concentrations lead to explant toxicity and severe browning, significantly reducing regeneration rates [44,45]. Therefore, maintaining a low bacterial density is a critical strategy in hairy root induction to protect host physiological activity and ensure sustained development.

A similar divergence is reflected in the selection of vacuum intensities between the two platforms, where transient expression utilized a fixed pressure of −0.07 MPa while stable hairy root induction was optimized at −0.06 MPa. This variation is directly governed by the distinct anatomical structures and tolerance thresholds of the respective target tissues. Transient transformation targets intact leaf tissues characterized by an extensive, air-filled spongy mesophyll network. Efficiently evacuating the gas from these large intercellular spaces requires a relatively higher negative pressure (−0.07 MPa) to drive full infiltration of the bacterial suspension across the mesophyll cell layers. Furthermore, the intact leaf epidermis, protected by a dense cuticle layer, provides sufficient mechanical resistance to tolerate this pressure differential without tissue collapse. Regarding hairy root induction, the system targets the shortened stem region where roots and leaves are mechanically excised. This mechanical wounding removes physical barriers but leaves the dense parenchymal cell target site highly vulnerable to structural compression. Applying excessive negative pressure (such as −0.07 MPa or above) to these wounded surfaces can trigger severe cell crushing, accelerated browning, and subsequent tissue necrosis, which severely suppresses long-term cell dedifferentiation and root organ primordia initiation. Consequently, maintaining a milder vacuum threshold of −0.06 MPa represents a critical parameter balance to successfully enhance bacterial tissue penetration while simultaneously preserving the physiological viability necessary for stable morphogenesis.

Regarding chemical stimulation, the contrasting interactive patterns between the transient and stable platforms provide deep insights into the tissue-specific tolerance of *A. mongolicum*. In the transient system, a significant interaction requires aligning high AS with low bacterial density to balance virulence activation and tissue vitality. Conversely, the hairy root platform displays a completely independent factor behavior where elevated AS levels consistently impair transformation success regardless of bacterial density. This generalized recalcitrance highlights that the exposed parenchymal cells of the shortened stem possess a substantially lower threshold for phenolic toxicity than intact leaf mesophyll cells. Excessive AS accumulation likely accelerates localized tissue scorching and suppresses the delicate cell dedifferentiation required for root primordia initiation. Consequently, bypassing the interactive dependency seen in leaves and maintaining an autonomously lower threshold of both bacterial titer (OD_600_ = 0.3) and phenolic application (100 μM) represents the optimal structural strategy to secure stable and reproducible morphogenesis in this wild monocot.

Although the optimized protocol establishes a reproducible transformation platform for *A. mongolicum*, it must be acknowledged that the peak hairy root induction rate achieved in this study (20.83%) remains modest and presents a practical constraint for immediate large-scale biotechnological configurations. This relatively low efficiency is deeply rooted in the inherent recalcitrance of the *Allium* genus. As typical monocots, their lack of a defined vascular cambium combined with rapid cell wall lignification severely restricts the number of wound-adjacent competent cells capable of integrating T-DNA and initiating organogenesis. To address this efficiency gap, a combination of biophysical and biochemical adjustments can be systematically evaluated in future studies.

Sonication-assisted *Agrobacterium*-mediated transformation (SAAT) offers a practical approach to facilitating bacterial penetration through the rigid physical barriers characteristic of desert monocots. As observed in *Papaver bracteatum* Lindl., the implementation of SAAT combined with specifically selected *A. rhizogenes* strains (such as C318) improved T-DNA delivery, helping to establish a stable hairy root culture system [46].

Additionally, the incorporation of non-ionic surfactants or membrane permeabilizers, such as Silwet L-77, Tween 20, or Triton X-100, could assist in modifying the infection interface. These agents can reduce the surface hydrophobicity of *A. mongolicum* tissues, encouraging a more uniform bacterial distribution across wound sites. In *Salvia miltiorrhiza* cultures, the use of such permeabilizers stimulated secondary metabolite exudation and altered host–pathogen interaction dynamics [47].

Furthermore, the chemical microenvironment during co-cultivation might be further optimized by exploring alternative virulence enhancers and metabolic modulators. Beyond acetosyringone, alternative plant-derived phenolic compounds—including vanillin, caffeic acid, p-coumaric acid, hydroxybenzoic acid, and gallic acid—can serve as exogenous signals to activate the *Agrobacterium* vir system [48]. Coordinating these phenolics with specific carbon sources, such as glucose or sucrose, may also modulate the bacterial metabolic state to indirectly support *vir* gene expression. Integrating these complementary strategies provides a reasonable pathway to steadily improve transformation rates in this recalcitrant species.

Beyond parameter optimization, the established *A. rhizogenes*-mediated hairy root induction platform holds distinct structural advantages for driving high-throughput functional genomics in *A. mongolicum.*

Regarding genome engineering, the system offers a practical application vector for CRISPR/Cas9-mediated gene editing in this recalcitrant monocot. Traditional whole-plant transformation and regeneration in the *Allium* genus remain extremely inefficient due to complex anatomical barriers and tissue recalcitrance. Because induced hairy roots can stably integrate exogenous T-DNA and proliferate indefinitely in vitro without exogenous phytohormones, they provide an ideal tissue matrix to bypass whole-plant regeneration hurdles. Downstream gene editing can be systematically executed by constructing single or multiplex gRNA cassettes driven by plant-endogenous promoters (such as U6/U3) alongside a codon-optimized Cas9 sequence. Following *A. rhizogenes* infection of the shortened stem, the resulting green-fluorescent independent lines can be rapidly harvested for genomic DNA extraction. Targeted sequencing or T7 Endonuclease I assays can then be deployed to quantify mutation efficiencies, allowing for the direct validation of gene-knockout lines at the cellular and root organ levels within a significantly compressed timeframe.

Simultaneously, this baseline platform is highly suitable for expanding specialized metabolite pathway analysis. *A*. *mongolicum* is rich in high-value secondary metabolites, including characteristic organosulfur volatiles, flavonoids, and polyphenols. However, functionally correlating specific biosynthetic genes (such as those in the alliin or phenylpropanoid pathways) with distinct chemical phenotypes has been historically constrained by the lack of transgenic tissues. Utilizing the optimized protocols established here, candidate genes can be either stably overexpressed or silenced via RNA interference (RNAi) within independent hairy root lineages. Because these clonal root lines maintain uniform genetic and biochemical backgrounds, they can be transferred into liquid culture systems for rapid biomass accumulation. Subsequent comparative metabolomic profiling using LC-MS or gas chromatography-mass spectrometry (GC-MS) will enable investigators to track precise alterations in metabolic flux. Quantifying the accumulation or depletion of specific intermediate compounds relative to non-transformed controls will effectively map complex metabolic networks and resolve the functional architecture of secondary metabolism unique to this desert species.

To properly contextualize the relative performance of the established *A. mongolicum* platform, it is helpful to compare it with transformation systems in related *Allium* crops, which are generally known to be difficult to transform via *Agrobacterium*. For transient expression, onion (*A. cepa*) epidermal cells often serve as a model; however, our vacuum-assisted whole-leaf infiltration system provides an alternative method for observing protein localization in planta within a wild relative. Regarding stable hairy root induction, our optimized efficiency of 20.83% represents a useful advancement for this uncultivated species. Quantitatively, this rate is lower than the highly optimized protocols reported in some cultivated relatives—such as the recent study in Welsh onion (*A. fistulosum*), which achieved 88.75% hairy root induction efficiency via stem disc infection [9]. However, it remains comparable to, and in some cases exceeds, traditional *Allium* transformation baselines that typically range from 5% to 15%. Considering the inherent structural barriers and higher tissue sensitivity of *A. mongolicum*, achieving a reproducible 20.83% induction rate provides a practical and workable foundation for future functional studies in this wild species.

It should be noted that the current study primarily evaluated the induction efficiency and early-stage phenotypic stability of the transgenic hairy roots over a period of approximately two months. The long-term growth kinetics, genetic stability during continuous subculturing, and, most importantly, the actual capacity of these root lines for the scalable production of *A. mongolicum*-specific secondary metabolites remain to be comprehensively characterized. These aspects, along with the scale-up in bioreactors, will be a primary focus of our future investigations, aiming to fully unlock the application potential of this desert species.

## 4. Materials and Methods

### 4.1. Bioinformatics Profiling of Candidate Proteins

Prior to experimental validation, in silico analysis of AmSWEET14 and AmJAZ2 was conducted. Conserved domain architectures were identified using the Batch Conserved Domain Search tool on the NCBI-CDD server (https://www.ncbi.nlm.nih.gov/Structure/cdd/wrpsb.cgi (accessed on 3 February 2026)). Theoretical subcellular localizations were predicted using the Plant-mPLoc server (http://www.csbio.sjtu.edu.cn/cgi-bin/PlantmPLoc.cgi (accessed on 3 February 2026)).

### 4.2. Construction of the Transient Expression System

#### 4.2.1. Seedling Cultivation and Explant Preparation for Transient Expression

Seeds of *A. mongolicum* (preserved at Inner Mongolia Agricultural University) were surface-sterilized with 75% ethanol for 30 s, 2% NaClO for 12 min, and rinsed six times with sterile water. Sterilized seeds were germinated on 1/2 Murashige and Skoog (MS) [49] solid medium (6 g/L agar) at 22 °C in the dark for 10 d, followed by 14 d under a 16-h light/8-h dark photoperiod to obtain axenic seedlings. Illumination was provided by cool-white fluorescent lamps positioned 25 cm above the cultures, with a light intensity of 20,000 Lux (corresponding to a calculated photosynthetic photon flux density of approximately 270 μmol m^−2^ s^−1^). To screen for receptive tissues, seedlings were sectioned into 0.5–1.0 cm segments of leaves, leaf sheaths, shortened stems (excised from the basal junction of roots and leaves), root bases, root mid-sections, and root tips.

#### 4.2.2. Plasmids and Bacterial Strains for Transient Expression

The pCAMBIA1302-GFP vector and recombinant plasmids pCAMBIA1302-AmJAZ2/AmSWEET14 were used. Additionally, nuclear (AtH2B-mRFP) and plasma membrane (AtSWEET11-mRFP) markers were utilized for co-localization assays. All plasmids were transformed into *Agrobacterium tumefaciens* GV3101.

#### 4.2.3. Media and Reagents for Transient Expression

1/2 MS medium: 2.21 g/L MS salts, 30 g/L sucrose, 6 g/L agar, pH 5.8.

Infection solution: 1/2 MS liquid medium with 10 mM MES, 10 mM MgCl_2_, and AS (evaluated at gradient levels of 100, 150, and 200 μM for parameter optimization, and fixed at 200 μM for subsequent formal assays), pH 5.8.

Co-cultivation medium: 1/2 MS solid medium with 10 mM MES, 10 mM MgCl_2_, AS (evaluated at gradient levels of 100, 150, and 200 μM for parameter optimization, and fixed at 200 μM for subsequent formal assays), and 6 g/L agar, pH 5.8.

#### 4.2.4. Exploration of Transient Infiltration and Efficiency Evaluation

Three methods were compared using GV3101 (OD_600_ = 0.4–0.8) harboring the empty pCAMBIA1302-GFP vector. *Agrobacterium* cells were inoculated into LB liquid medium supplemented with Kanamycin (50 mg/L) and Rifampicin (50 mg/L) to maintain the binary vector and helper plasmid, respectively. The culture was incubated at 28 °C with constant shaking at 200 rpm until the optical density (OD_600_) reached approximately 0.8–1.0. Subsequently, the bacterial cells were harvested by centrifugation at 4000× *g* for 10 min at 4 °C. The supernatant was completely discarded to remove residual medium and antibiotics. The bacterial pellet was resuspended in the infection solution and adjusted to OD_600_ values of 0.4, 0.6, and 0.8 to evaluate the transformation efficiency. Based on the optimization results, an OD_600_ of 0.4 was determined to be the optimal concentration and was selected for all subsequent subcellular localization assays.

Vacuum impregnation: Leaves were immersed in the suspension and subjected to −0.07 MPa for 10 min.

Cut-and-soak method: Micro-wounds were made with sterile scissors dipped in infection solution, followed by wrapping with saturated sterile cotton for 5 min.

Syringe infiltration: Suspensions were slowly injected into the leaf interior using a 1 mL syringe with a 34 G needle (0.18 mm O.D.).

GFP signals were observed using a confocal laser scanning microscope (C2 PLUS, Nikon, Tokyo, Japan) after 2 d. For GFP fluorescence imaging, a 488 nm excitation laser was used with an emission window of 500–530 nm. For mRFP fluorescence imaging, a 561 nm excitation laser was used with an emission window of 570–620 nm. To optimize the signal-to-noise ratio and prevent signal saturation, the detector high voltage (HV) was set to 110, and the offset was maintained at −20 for image acquisition. For each biological replicate, 30 individual seedlings were treated per group. The transient transformation efficiency was calculated by counting the number of fluorescent leaves:

(1) $$Transformation\;Efficiency\left(\%\right)=\frac{Number\;of\;leaves\;with\;GFP\;signals}{Total\;number\;of\;treated\;leaves}\times 100\%$$

#### 4.2.5. Transient Expression in *Nicotiana benthamiana* and Co-Localization

Healthy and uniform *N. benthamiana* plants (four to six weeks old) were employed for transient expression studies. *Agrobacterium* cultures (OD_600_ = 0.8) carrying target proteins and mRFP markers were mixed equally and infiltrated into the abaxial leaf surface. An infiltration volume of approximately 0.5 mL was applied to each leaf to ensure complete permeation of the leaf lamina. Co-localization was observed 48–72 h post-infiltration.

### 4.3. Establishment of the Hairy Root Induction System

#### 4.3.1. Explant Preparation for Hairy Root Induction

Seedlings were cultivated as in Section 4.2.1. Twenty-four-day-old seedlings were sectioned into 0.5–1.0 cm segments of roots, shortened stems, and leaves for induction assays.

#### 4.3.2. Plasmids and Bacterial Strains for Hairy Root Induction

The *A. rhizogenes* strains utilized in this study—K599, LBA9402, and Ar.qual—were all wild-type, agropine-type strains harboring their native, non-disarmed Ri-plasmids. These strains were used to co-transform the host plant with the endogenous Ri T-DNA (responsible for hairy root induction) and the binary vector T-DNA. All strains were engineered to carry the recombinant binary vector pCAMBIA1302-GFP.

#### 4.3.3. Media and Reagents for Hairy Root Induction

Infection solution: 1/2 MS liquid with 2.5 mM MES and 100 μM AS, pH 5.8.

Co-cultivation medium: 1/2 MS solid with 2.5 mM MES, 100, 150, and 200 μM AS (according to the orthogonal experimental design), and 6 g/L agar, pH 5.8.

Selection medium: 1/2 MS solid with 200 mg/L Timentin, 20 mg/L Hygromycin, and 6 g/L agar, pH 5.8.

#### 4.3.4. Hairy Root Induction and Induction Rate Evaluation

*Agrobacterium* cells were inoculated into an LB liquid medium supplemented with Kanamycin (50 mg/L) and Rifampicin (50 mg/L) to maintain the binary vector and helper plasmid, respectively. The culture was incubated at 28 °C with constant shaking at 200 rpm until the optical density (OD_600_) reached approximately 0.8–1.0. Subsequently, the bacterial cells were harvested by centrifugation at 4000× *g* for 10 min at 4 °C. The supernatant was completely discarded to remove the residual medium and antibiotics. The bacterial pellet was resuspended in the infection solution and adjusted to OD_600_ values of 0.3, 0.5, and 0.7 for the transformation optimization experiments. Following the parameter evaluation, an OD_600_ of 0.3 was determined as the optimal concentration and applied for all formal hairy root induction assays. Explants were subjected to vacuum infiltration at −0.06 to −0.08 MPa for 10 min. Following co-cultivation (23 °C, dark, 60% relative humidity, 2–4 d), explants were washed and transferred to a selection medium for 14 d. Each biological replicate contained 40 independent explants. The induction rate was calculated as follows:

(2) $$Induction\;Efficiency\;\left(\%\right)=\frac{Number\;of\;explants\;producing\;hairy\;roots}{Total\;number\;of\;inoculated\;explants}\times 100\%$$

#### 4.3.5. PCR Verification

Total genomic DNA was isolated with a TIANGEN kit (Beijing, China) according to the manufacturer’s instructions. Specific primers provided in Table S1 were utilized to amplify the GFP gene to verify binary vector integration. To confirm the co-transformation of the native Ri T-DNA and rule out false positives, multiplex and counter-screening PCRs were performed using primers targeting the *rolB* gene (an Ri-plasmid T-DNA marker) and the *virD2* gene (an *Agrobacterium*-specific backbone marker). PCR conditions were: 94 °C for 10 min; 30 cycles (94 °C for 30 s, 53 °C for 30 s, 72 °C for 90 s); 72 °C for 5 min. Products were analyzed via 2% agarose gel electrophoresis.

### 4.4. Experimental Design and Statistical Analysis

An orthogonal experimental design was employed to systematically optimize transformation parameters. To analyze the baseline data, a Multi-factor Analysis of Variance (Multi-factor ANOVA) via the General Linear Model (GLM) framework was implemented to evaluate the statistical significance of individual main effects as well as their potential factor interactions (e.g., bacterial density × AS concentration). Multiple comparisons among treatment means were performed using Tukey’s honest significant difference (HSD) post hoc test (*p* < 0.05) with a sample size of *n* = 3 independent biological replicates. Range analysis (including average efficiency k and range R) was concurrently conducted to rank the order of factor influence. All statistical operations were executed using SPSS 22.0. Data are presented as the Mean ± Standard Deviation (SD). Range analysis (R) and average efficiency (k) were used to evaluate the influence of each factor. All experiments were performed with three biological replicates.

## 5. Conclusions

In this study, two optimized genetic transformation systems—a transient expression platform and a stable hairy root induction system—were successfully established for the desert plant *A. mongolicum*. For subcellular localization, vacuum impregnation of leaf tissues was identified as the optimal method (OD_600_ = 0.4, AS = 200 μM). Its biological applicability was evaluated using the *AmJAZ2* and *AmSWEET14* genes, where their specific subcellular localizations in *A. mongolicum* were inferred from the GFP spatial patterns and robustly supported by formal co-localization assays in *N. benthamiana*. For hairy root induction, infecting shortened stems with the *A. rhizogenes* strain Ar.qual proved most effective. The optimal induction parameters were determined as OD_600_ = 0.3, AS = 100 μM, vacuum infiltration (–0.06 MPa for 10 min), and a 72-h co-cultivation period. The stable integration and expression of the transgene in the hairy roots were definitively confirmed via GFP fluorescence and PCR analysis. This research provides baseline genetic transformation protocols for *A. mongolicum*, establishing a primary technical report that may support subsequent functional genomics, secondary metabolism studies, and long-term molecular breeding efforts in this species.

## Figures and Tables

**Table 1:** Optimization of *Agrobacterium*-mediated transient transformation conditions in *A. mongolicum* using an orthogonal design.

Treatment (No.)	OD600 (Factor A)	AS Concentration (Factor B)	Transformation Efficiency (%)
1	0.4	200	75.56 ± 5.09 a
2	0.4	150	63.33 ± 3.33 ab
3	0.4	100	56.67 ± 3.33 bc
4	0.6	100	55.56 ± 5.09 bc
5	0.6	150	50.00 ± 3.33 bcd
6	0.6	200	44.44 ± 5.09 cde
7	0.8	100	41.11 ± 5.09 cde
8	0.8	200	35.56 ± 5.09 de
9	0.8	150	34.44 ± 8.39 e
k1	65.18	51.11	
k2	50.00	49.26	
k3	37.04	51.85	
R	28.15	2.60	

Notes: k1, k2, k3 represent the mean transformation efficiency for each factor at levels 1, 2, and 3, respectively. R represents the range (the difference between the maximum and minimum k-values within a single factor). Data in the efficiency columns are expressed as Mean ± SD (*n* = 3). Different lowercase letters within the same column indicate statistically significant differences among treatment combinations according to Tukey’s HSD test (*p* < 0.05).

**Table 2:** Orthogonal experimental design and results for optimizing hairy root induction in *A. mongolicum.*

Treatment (No.)	OD_600_ (Factor A)	AS Concentration (Factor B)	Induction Efficiency (%)
1	0.3	100	18.33 ± 1.44 a
2	0.3	150	13.33 ± 1.44 bc
3	0.3	200	12.50 ± 2.50 c
4	0.5	100	15.00 ± 2.50 ab
5	0.5	150	8.33 ± 1.44 d
6	0.5	200	8.33 ± 1.44 d
7	0.7	100	7.50 ± 2.50 de
8	0.7	150	5.83 ± 1.44 e
9	0.7	200	5.00 ± 2.50 e
k1	14.72	13.61	
k2	10.55	9.16	
k3	6.11	8.61	
R	8.61	5.00	

Notes: Data in the efficiency columns are expressed as Mean ± SD (*n* = 3). Different lowercase letters within the same column indicate statistically significant differences among treatment combinations according to Tukey’s HSD test (*p* < 0.05).

**Table 3:** Optimization of co-cultivation time and vacuum intensity for *A. mongolicum* hairy root induction.

Treatment (No.)	Co-Cultivation Time (Factor A)	Vacuum Pressure (MPa, Factor B)	Induction Efficiency (%)
1	48 h	−0.04	9.17 ± 1.44 def
2	48 h	−0.06	16.67 ± 1.44 abc
3	48 h	−0.08	14.17 ± 1.44 bcd
4	72 h	−0.04	17.50 ± 2.50 ab
5	72 h	−0.06	20.83 ± 1.44 a
6	72 h	−0.08	8.33 ± 2.89 ef
7	96 h	−0.04	10.83 ± 1.44 cde
8	96 h	−0.06	8.33 ± 1.44 ef
9	96 h	−0.08	6.67 ± 1.44 f
k1	13.34	12.50	
k2	15.55	15.28	
k3	8.61	9.72	
R	6.94	5.55	

Notes: Data in the efficiency columns are expressed as Mean ± SD (*n* = 3). Different lowercase letters within the same column indicate statistically significant differences among treatment combinations according to Tukey’s HSD test (*p* < 0.05).

## Data Availability

Data will be made available on request.

## Supplementary Materials

The following supporting information can be downloaded at: https://www.mdpi.com/article/10.3390/plants15121799/s1, Table S1: List of the primers used in the study. Figure S1. Bioinformatics prediction and conserved domain analysis of AmSWEET14 and AmJAZ2.
