# Supplementary material for: Establishment of Transient Expression and Hairy Root Induction Systems in Allium mongolicum
Source: Plants (Basel). 2026 Jun 11;15(12):1799. doi: 10.3390/plants15121799 (PMC13306547; doi:10.3390/plants15121799)
Supplement: Supplementary file 1 [file plants-15-01799-s001.zip › Supplementary Materials Figure S1.pdf]

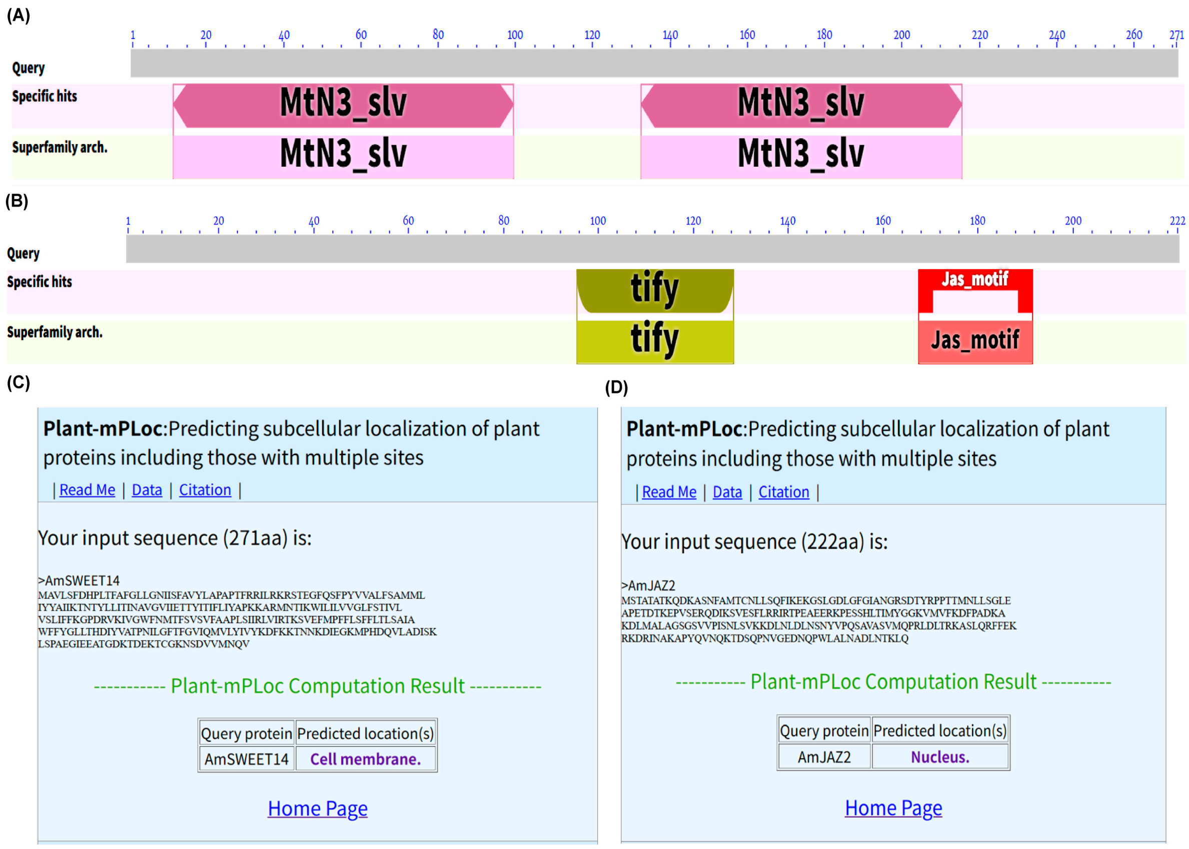

Figure S1. Bioinformatics prediction and conserved domain analysis of AmSWEET14 and AmJAZ2. (A) Conserved domain architecture of AmSWEET14 showing two characteristic duplicated MtN3_slv superfamily domains. (B) Conserved domain architecture of AmJAZ2 containing the typical TIFY domain and the C-terminal Jas_motif. (C–D) Theoretical subcellular localization predictions for AmSWEET14 (C) and AmJAZ2 (D) executed via the online Plant-mPLoc server.
